# ADAR1 promotes systemic sclerosis *via* modulating classic macrophage activation

**DOI:** 10.3389/fimmu.2022.1051254

**Published:** 2022-12-01

**Authors:** Chenming Sun, Dunpeng Cai, Shi-You Chen

**Affiliations:** ^1^ Department of Pathogenic Microbiology and Immunology, School of Basic Medical Sciences, Xi’an Jiaotong University, Xi’an, Shaanxi, China; ^2^ Institute of Infection and Immunity, Translational Medicine Institute, Xi’an Jiaotong University Health Science Center, Xi’an, Shaanxi, China; ^3^ Xi’an Key Laboratory of Immune Related Diseases, Xi’an, Shaanxi, China; ^4^ Departments of Surgery, University of Missouri School of Medicine, Columbia, MO, United States; ^5^ Department of Medical Pharmacology and Physiology, University of Missouri School of Medicine, Columbia, MO, United States

**Keywords:** ADAR1, macrophage activation, systemic sclerosis, NF-κB, IL-1β

## Abstract

**Introduction:**

As a multisystem autoimmune disorder disease, systemic sclerosis (SSc) is characterized by inflammation and fibrosis in the skin and other internal organs. However, mechanisms underlying the inflammatory response that drives the development of SSc remain largely unknown.

**Methods:**

ADAR1 heterozygous knockout (AD1+/-) mice and myeloid-specific ADAR1 knockout mice were used to determine the function of ADAR1 in SSc. Histopathological analyses and western blot confirmed the role of ADAR1 in bleomycin-induced increased skin and lung fibrosis.

**Results:**

In this study, we discover that adenosine deaminase acting on RNA (ADAR1), a deaminase converting adenosine to inosine (i.e., RNA editing) in RNA, is abundantly expressed in macrophages in the early stage of bleomycin-induced SSc. Importantly, ADAR1 is essential for SSc formation and indispensable for classical macrophage activation because ADAR1 deficiency in macrophages significantly ameliorates skin and lung sclerosis and inhibits the expression of inflammation mediator inducible NO synthase (iNOS) and IL-1β in macrophages. Mechanistically, deletion of ADAR1 blocks macrophage activation through diminishing NF-κB signaling.

**Discussion:**

Our studies reveal that ADAR1 promotes macrophage activation in the onset of SSc. Thus, targeting ADAR1 could be a potential novel therapeutic strategy for treating sclerosis formation.

## Introduction

Systemic sclerosis (SSc), as a multisystem autoimmune disease, is characterized by fibrosis of the skin and other organs, like lung, heart, kidney, and gastrointestinal tract, which is accompanied by abnormalities in innate and adaptive immunity (1, 2). Although the etiology remains ambiguous, several advances suggest that SSc is closely associated with the immune response, including immunological activation and tissue infiltration of innate immune cells, microvascular endothelium injury by activated macrophages, and fibroblast activation by proinflammatory cytokines, which leads to excessive deposition of extracellular matrix (3, 4). Recent studies highlight that macrophage infiltration is a prerequisite for SSc, as evidenced by the bleomycin-induced dermal and lung fibrosis in nude, rag-deficient, or SCID mice (5–7). Although these immunodeficient transgenic mice do not have mature T cells, bleomycin induces pathological features of SSc, suggesting that macrophages alone are sufficient to initiate the onset of SSc. However, the mechanisms by which macrophages drives the development of SSc remain largely unknown.

As the effector and regulator cells in the immune system, macrophages display diverse plasticity and physiology, which give rise to distinct populations with different functions. The M1 macrophages, which also named classically activated macrophages, are stimulated by interferon-gamma (IFNγ) and lipopolysaccharides (LPS). This divergent population has a robust capability of producing high levels of pro-inflammatory cytokines including interleukin-1β (IL-1β) and nitric oxide which specifically depends on inducible nitric oxide synthase (iNOS) expression through NF-κB signaling (8–10). M1 macrophages contribute to the pathogenesis of SSc. Previous studies have shown that pro-inflammatory macrophages and cytokines, such as IL-1β and TNFα, are significantly higher in the bronchoalveolar lavage fluid and peripheral blood of SSc patients (11). Additionally, SSc patients show boosted NF-κB activity (12) and augmented expression of a cluster of IFN-regulated genes (13). Despite a broad appreciation of SSc being an inflammation-related disease, the mechanisms regulating macrophage function in SSc pathogenesis has not been sufficiently addressed. On the other hand, alternatively activated macrophages (M2 macrophages), as stimulated by IL-4, have the ability to promote arginase activity and contribute to tissue fibrosis through secreting pro-fibrogenic cytokines such as TGF-β, PDGF and CCL18. Blocking the functions of these cytokines with the neutralizing antibodies has been shown to reverse the pro-fibrogenic function of M2 macrophages (14–16). During the development of human SSc, M2 macrophages are abundantly observed in the skins of SSc patients (2). Therefore, both M1 and M2 macrophages are involved in SSc.

RNA editing, particularly the conversion of adenosine to inosine (A-to-I), is one of the posttranscriptional mechanisms for gene expression (17). A-to-I editing is catalyzed by adenosine deaminase acting on RNAs (ADARs). Among three members (ADAR1-3) identified in mammalian cells, ADAR1 has been shown to display most versatile roles in biological and pathological conditions (18–20). ADAR1-mediated A-to-I RNA editing alters RNA structure and gene coding sequence of proteins (21). Recent studies have extended our knowledge on editing-independent mechanisms of ADAR1 such as RNA binder (22, 23).

In skin biopsies and peripheral blood of SSc patients, an increased level of ADAR1 is observed (24). However, the potential role of ADAR1 in SSc has not been reported. Although ADAR1 has been found to be involved in inflammatory response, the current results remain controversial. ADAR1 expression is increased in mice with systemic inflammation after endotoxin treatment (25). ADAR1 inhibition attenuates local inflammation due to acute lung injury (26). These studies suggest that ADAR1 favors inflammatory response. However, a few other studies suggest an anti-inflammatory function of ADAR1. For instance, inhibiting ADAR1 increases inflammatory cytokine levels in experimental septic model or liver injury model (27–29). In addition, in viral infection, ADAR1 dampens macrophage activation *via* blocking viral replication and suppressing IFN signaling (30). Also, overexpression of ADAR1 suppresses inflammatory cytokine expression in the RAW264.7 macrophages (28). Therefore, elucidating ADAR1 function in modulating macrophages, especially the key inflammatory mediator M1 macrophages, in SSc development would shed considerable new light on the roles of ADAR1 in inflammation and diseases.

In the present study, we have identified a novel role of ADAR1 in promoting pathogenesis of the bleomycin-induced SSc. ADAR1 deficiency in macrophages significantly ameliorates skin and lung sclerosis. Mechanistically, ADAR1 mediates bleomycin-induced M1 macrophage activation and the iNOS and IL-1β production through enhancing NF-κB signaling.

## Materials and methods

### Mice

ADAR1 knockout mice (B6.129(Cg)-Adartm1.1Phs/KnkMmjax), ADAR1fl/fl mice (B6.129-Adartm1Knk/Mmjax), and LysM-cre mice (B6.129P2-Lyz2tm1(cre)Ifo/J) were purchased from the Jackson Laboratory (Bar Harbor, ME). Mice aged 6-8 weeks were used for analyses in this study. All mice were housed under conventional conditions in the animal care facilities by the Xi’an Jiaotong University Division of Laboratory Animal Research. All animal procedures were approved by the Institutional Animal Care and Use Committee of Xi’an Jiaotong University, Xi’an Center for Disease Control.

### Cytokines and reagents

Bleomycin was purchased from ThermoFisher Scientific. The following antibodies were used in Western blot and immunofluorescent staining. ADAR1 (D-8) and collagen type Ia1 (COL1a1) (D-13) and Lamin B (C-20) were obtained from Santa Cruz Biotechnology. iNOS (4E5) were purchased from Abcam. IL-1 b (3A6), NF-κB p65 (D14E12), phospho- NF-κB p65 (Ser536) were from Cell Signaling Technology. GAPDH antibody was from Proteintech, and F4/80 (BM8) antibody was from BioLegend. Nuclei were stained with DAPI (Vector Laboratories). The secondary antibodies were from Cell Signaling Technology. M-CSF and IFNγ were purchased from R&D Systems. M-CSF was used at 10 ng/ml, and IFNγ was used at 100 ng/ml. LPS was obtained from Sigma-Aldrich (St. Louis, MO) and used at 100 ng/ml.

### Bleomycin-induced murine model of SSc

To induce skin fibrosis, bleomycin (0.02U) dissolved in 50uL PBS was injected subcutaneously (s.c.) into a single location on the back of mice daily for 28 days. To induce pulmonary fibrosis, bleomycin (0.2 U) in 100 uL PBS was applied intranasally once, and the mice were euthanized 24 days later. PBS was used as control in both models. The skin or lung tissues were collected for further analyses.

### Histopathology and immunofluorescent staining

Skin and lung tissues were fixed in 4% paraformaldehyde (PFA) and embedded in paraffin. Tissue sections (5 µm thick) were stained with hematoxylin-eosin (H&E) or Masson’s trichrome using commercial kits (Dako) for histopathological analyses according to the manufacturer’s protocol. For immunofluorescent staining, serial sections (10 µm) from OCT-embedded frozen tissues or primary cultured cells were fixed in cold acetone or 4% paraformaldehyde. After blocking with 1% goat serum, sections were incubated with primary antibodies at room temperature for 2 hours followed by incubation with fluorescent dye-conjugated secondary antibodies for 1 hour. Images were acquired with a fluorescence microscope (Nikon Instruments Inc).

### Isolation and cell culture of peritoneal macrophages and bone marrow-derived macrophages

Mouse PEMs were isolated from the peritoneum of mice as previously described (31). Briefly, peritoneal cells were harvested by injecting 10 ml of PBS (5 injections, 2mL each) into the peritoneal cavity. 1-2×10^6^ cells were collected from one mouse, in which 0.5×10^6^ cells were macrophages. After flushing with cold PBS, the cells were diluted to 1×10^6^ cells/ml in Dulbecco’s Modified Eagle’s medium (DMEM) supplemented with 10% heat-inactivated fetal bovine serum (FBS) and incubated in 12-well plates in a humidified CO_2_ incubator at 37°C for 2 hours. The non-adherent cells were removed by washing with warm PBS. More than 90% of the adherent cells were macrophages.

Bone marrow cells were used to generate BMDMs as previously described (2). Bone marrow was aseptically flushed out from the tibiae and femurs of mice and depleted of red blood cells using red blood cell lysis buffer (Roche Corporation). After re-suspended in DMEM medium, the cells were placed in a cell culture dish and incubated at 37°C for 2 hours to remove adherent cells. The non-adherent cells were re-suspended in DMEM medium supplemented with 10% heat-inactivated FBS, 100 IU/ml penicillin, 100 µg/ml stereptomycin, 2 mM L-Glutamine (Thermo Fisher Scientific), and 10 ng/ml M-CSF and cultured for 7 days. Non-adherent cells were removed, and the M-CSF-conditioned medium was changed on day 3 and day 5. To acquire the M1 macrophages, 100 ng/ml IFNγ and 100 ng/ml LPS were used to stimulate the macrophages for 3 hours for mRNA expression or 6 hours for protein assays.

### Quantitative reverse transcription PCR

The RNeasy Mini Kit (QIAGEN) was used to extract total RNA from cells or tissues according to the manufacturer’s instruction. Complementary DNA (cDNA) was synthesized using cDNA Synthesis Kit (TOYOBO). qPCR was performed based on the StepOnePlus Real-Time PCR System (Thermo Fisher Scientific) using SYBR Green RT-qPCR Master Mix (GenStar).

### Western blotting

PEMs, BMDMs, or tissues were lysed in RIPA lysis buffer (1% Nonidet P-40, 0.1% sodium dodecyl sulfate (SDS), 0.5% sodium deoxycholate, 1 mM sodium orthovanadate, and protease inhibitors) to extract the total proteins. Samples were separated on SDS-polyacrylamide gels and electro-transferred onto nitrocellulose membranes (Amersham Biosciences). After blocking with 5% BSA, the membranes were incubated with various primary antibodies at 4°C overnight, followed by incubation with secondary antibodies at room temperature for 1 hour. The protein expression was measured by FUSION Solo.6 s (Vilber).

### Statistical analysis

Statistical analyses were applied to biologically independent mice or technical replicates for each experiment. All experiments were independently repeated at least three times. All data are presented as the mean + SD. One-way or two-way ANOVA was used for comparison among different groups. All bar graphs include means with error bars to show the distribution of the data. The level of significance is indicated as *P < 0.05; **P < 0.01; ***P < 0.001.

## Results

### ADAR1 is essential for the development of SSc

**Figure 1:**
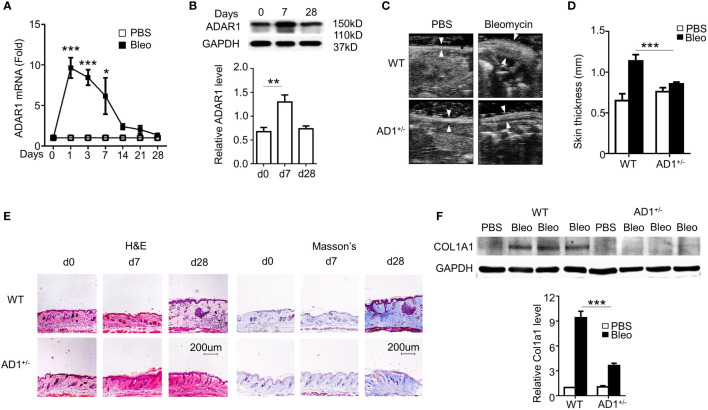
ADAR1 was essential for the pathogenesis of systemic sclerosis (SSc). **(A, B)** Mice were injected s.c. with bleomycin (Bleo: 0.02U) for the times indicated. ADAR1 mRNA **(A)** and protein **(B)** expression in skin were detected by qPCR and Western blotting, respectively. **(B)** ADAR1 protein levels were quantified by normalizing to GAPDH (n = 6). *P<0.05, **P<0.01, ***P<0.001 vs PBS-treated group for each isoform. 1-way ANOVA was used for comparison among groups. **(C)** ADAR1 heterozygous deletion (AD1^+/-^) attenuated bleomycin-induced skin fibrosis compared with WT mice. Skin sections were collected 28d after bleomycin injection, and skin thickness was measured by ultrasonography and indicated by white arrows **(D)** Quantification of the skin thickness in bleomycin-treated WT and AD1^+/-^ mice compared with PBS control (n = 6). ***P<0.001. **(E)** ADAR1 deletion inhibited bleomycin-induced collagen deposition in skin, as shown by Masson’s trichrome staining. H&E staining showed skin structure. 20x power of objective lens was used to acquire images. **(F)** ADAR1 deletion blocked bleomycin-induced Col1a1 expression in skin tissues, as determined by Western blotting. COL1A1 protein levels were quantified by normalizing to GAPDH (n = 6). ***P<0.001. 2-way ANOVA was used for comparison among groups.

Bleomycin-induced skin fibrosis in mice was used to study SSc (2). Along with SSc progression, ADAR1 mRNA (**Figure 1A**) and protein expression (**Figure 1B**) of both isoforms (p150 and p110) (21) was significantly upregulated in the skin tissues. A substantial ADAR1 expression was induced as early as day 1 following the bleomycin injection, suggesting that ADAR1 may be involved in SSc formation.

Due to embryonic lethality of homozygous ADAR1 mutant, ADAR1 heterozygous knockout (AD1+/-) mice were used to determine the function of ADAR1 in SSc. As the hallmark of skin fibrosis, WT mice with bleomycin injection for 28 days developed significant thickened dermis that results from collagen deposition. However, ADAR1 deletion remarkably reduced the skin thickening as measured by ultrasound imaging (**Figures 1C, D**). Histopathological analyses using H&E staining of the skin sections further confirmed that ADAR1 deficiency effectively attenuated bleomycin-induced increased skin thickness (**Figure 1E**, left panels). As fibrosis is characterized by excessive collagen deposition, Masson’s trichrome staining was used to detect collagen accumulation in the bleomycin challenged skins. As shown in **Figure 1E** (right panel), the collagen deposition was markedly attenuated in ADAR1-deficient mouse skin tissues compared to the WT after bleomycin injection for 28 days. Consistently, the collagen protein COL1A1 expression was also decreased considerably in bleomycin-treated AD1+/- mice (**Figure 1F**). Taken together, these data demonstrated that ADAR1 is essential for the SSc development.

### Macrophage ADAR1 is essential for SSc

**Figure 2:**
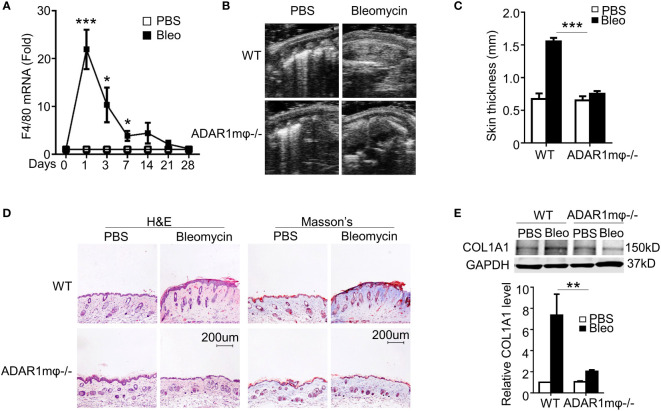
ADAR1 deficiency in macrophages (ADAR1mφ^-/-^) ameliorated skin sclerosis formation. **(A)** Mice were injected s.c. with bleomycin (Bleo: 0.02U) for the times indicated. Total RNA was extracted from skin tissues, and F4/80 mRNA levels were detected by RT-qPCR. *P<0.05, ***P<0.001 vs PBS-treated group for each isoform. **(B)** ADAR1mφ^-/-^ attenuated bleomycin-induced skin fibrosis compared with WT mice. Skin sections were collected 28d after bleomycin injection, and skin thickness was measured by ultrasonography and indicated by arrows. **(C)** Quantification of the skin thickness in bleomycin-treated WT and ADAR1mφ^-/-^ mice. ***P<0.001 vs. PBS control, n = 6. **(D)** ADAR1mφ^-/-^ inhibited bleomycin-induced collagen deposition in skin, as shown by Masson’s trichrome staining. H&E staining showed skin structure. 20x power of objective lens was used to acquire the images. **(E)** ADAR1 deletion blocked bleomycin-induced COL1A1 expression in skin tissues, as determined by Western blotting. COL1A1 protein levels were quantified by normalizing to GAPDH. **P<0.01, n = 6. 2-way ANOVA was used for comparison among groups.

In agreement with previous report that macrophage infiltration is one of the essential factors initiating SSc (32), we observed a rapid and remarkable increase in mRNA expression of macrophage marker F4/80 in bleomycin-treated skin tissues of C57BL/6 mice (**Figure 2A**). Notably, the highest F4/80 level was concomitant with ADAR1 expression, indicating that ADAR1 may promote bleomycin-induced SSc by modulating macrophage function.

To determine if ADAR1 in macrophages is essential for SSc formation, we generated myeloid-specific ADAR1 knockout mice (ADAR1mφ-/-) by crossing ADAR1-floxed mice with LysM-cre mice. After bleomycin injection for 28 days, ADAR1mφ-/- mice exhibited much less skin thickness compared to WT mice as measured by ultrasonography (**Figures 2B, C**). Moreover, both the collagen deposition and protein expression were markedly reduced in ADAR1mφ-/- mouse skin tissues under bleomycin treatment (**Figures 2D, E**).

### Macrophage ADAR1 is essential for bleomycin-caused lung fibrosis

**Figure 3:**
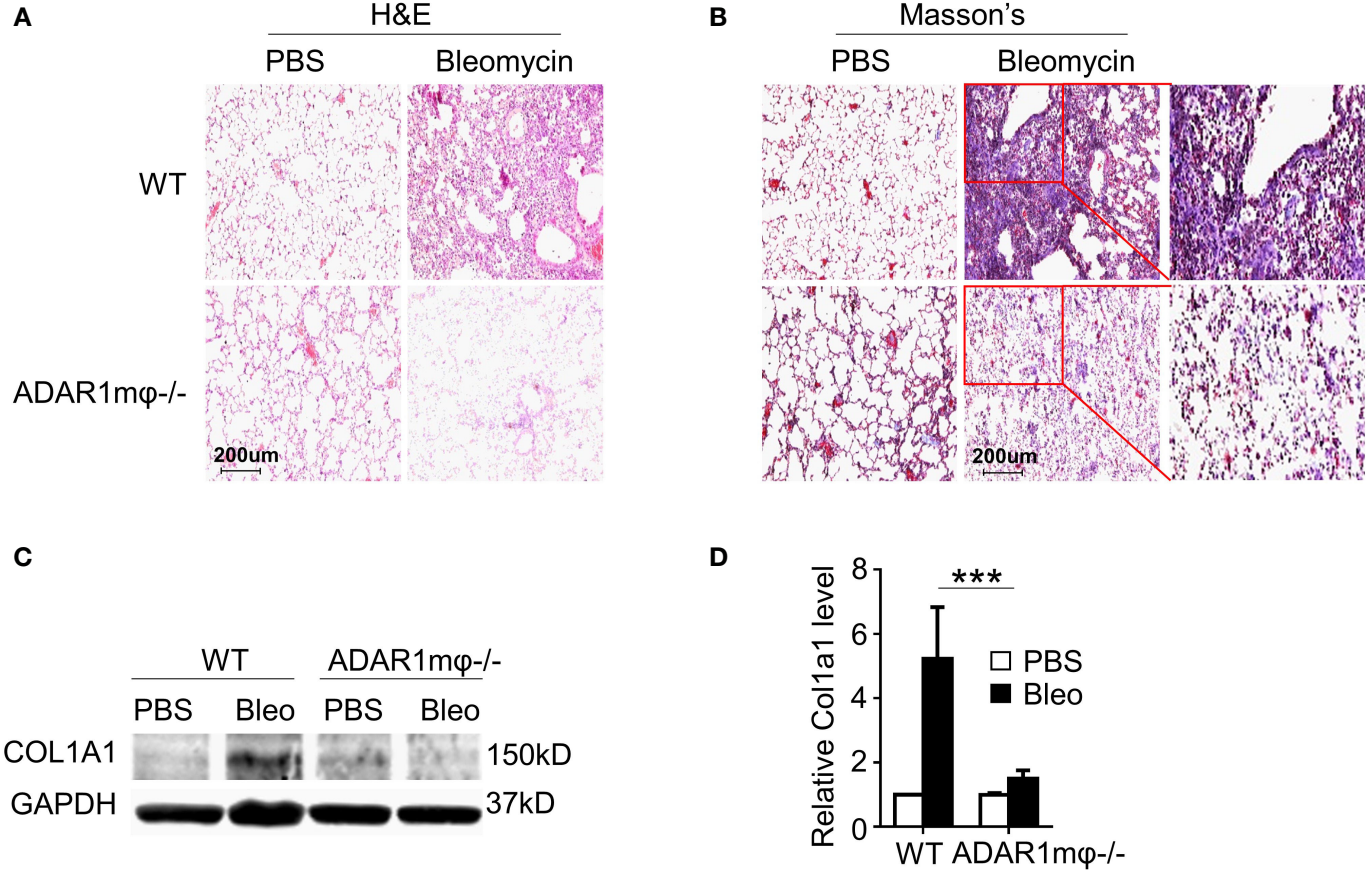
ADAR1mφ^-/-^ blocked bleomycin induced lung fibrosis. Lung fibrosis was induced in WT and ADAR1mφ^-/-^ mice by bleomycin injection (0.2U) for 24 days. **(A, B)** ADAR1mφ^-/-^ inhibited bleomycin-induced structure damage and collagen deposition in lung as shown by H&E staining **(A)** and Masson’s trichrome staining, respectively. 20x power of objective lens was used to acquire images. **(C)** ADAR1mφ^-/-^ blocked bleomycin-induced COL1A1 expression in lung tissues, as determined by Western blotting. **(D)** COL1A1 protein levels in C were quantified by normalizing to GAPDH. ***P < 0.001, n = 6. 2-way ANOVA was used for comparison among groups.

Pulmonary lesions are the leading cause of death in patients with SSc (33). Thus, we sought to determine whether ADAR1 plays a role in lung sclerosis. Lung fibrosis was induced by bleomycin injection in WT and ADAR1mφ-/- mice. As analyzed by H&E staining, the bleomycin-caused lung structure damage was remarkably alleviated in ADAR1mφ-/- mice (**Figure 3A**). Furthermore, bleomycin-treated ADAR1mφ-/- mice exhibited a significant reduction in collagen deposition and protein expression in the lung compared with WT mice (**Figures 3B–D**). These data suggested that ADAR1 promote systemic sclerosis *via* the modulation of macrophage function.

### ADAR1 promotes the macrophage inflammation in SSc

**Figure 4:**
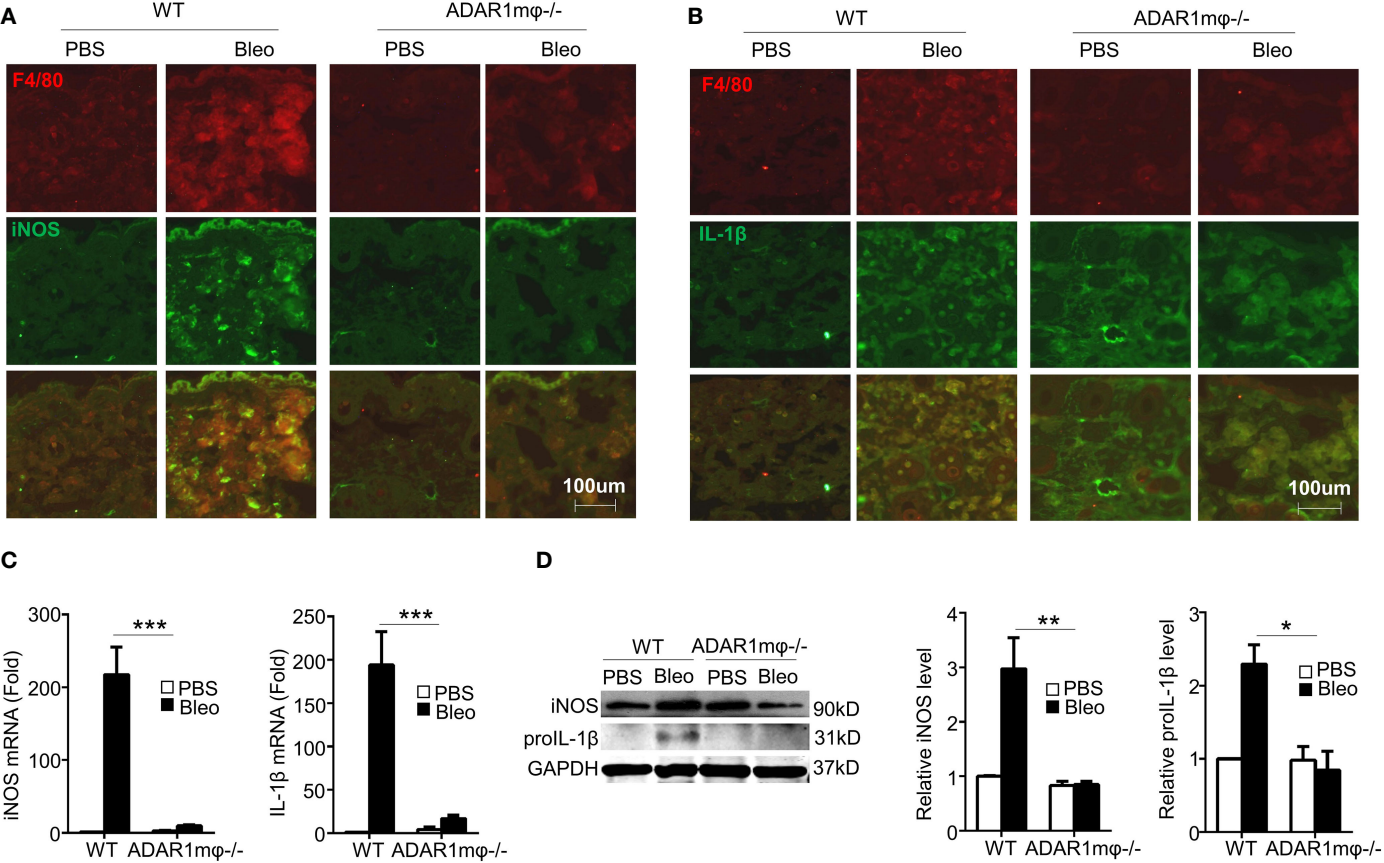
Macrophage ADAR1 deficiency (ADAR1mφ^-/-^) modulated inflammation in bleomycin induced skin sclerosis. Mice were injected s.c. with bleomycin (Bleo: 0.02U) for 1 day. **(A, B)** Frozen skin sections were co-immunostained with F4/80 and iNOS antibodies **(A)** or IL-1β antibodies **(B)**. 20x power of objective lens was used to acquire images. **(C, D)** ADAR1mφ^-/-^ attenuated the mRNA and protein expression of iNOS and pro-inflammatory cytokines IL-1β. The mRNA **(C)** and protein **(D)** levels of iNOS and IL-1β in bleomycin-treated mouse skin were quantified by qPCR and Western blot, respectively. And iNOS and IL-1β protein levels were normalized to GAPDH. Data shown are the mean ± SD of three independent experiments. *P < 0.05, **P < 0.01, ***P < 0.001 (ADAR1mφ^-/-^ vs WT mice with bleomycin injection). 2-way ANOVA was used for comparison among groups.

Inflammation is essential in driving the pathogenesis of SSc (34). Pro-inflammatory cytokines, such as IL-1β and nitric oxide production, were upregulated in sclerosis tissues (35, 36). Since macrophage ADAR1 is essential for sclerosis formation (**Figure 2**), we sought to investigate the relation of ADAR1-expressing macrophages and the inflammatory response in bleomycin-induced skin fibrosis. Immunostaining of skin sections showed that iNOS (**Figure 4A**) and IL-1β (**Figure 4B**) were co-localized with macrophage marker F4/80. These observations strongly supported that pro-inflammatory (M1) macrophages, the major inflammation mediator, play a critical role in bleomycin-induced SSc development. Importantly, ADAR1mφ-/- mice showed a remarkably lower inflammation in the skins after bleomycin treatment, as evidenced by the declined mRNA and protein expression of iNOS and IL-1β (**Figures 4C, D**), which correlated with the attenuated SSc formation and progression in ADAR1mφ-/- mice (**Figure 2**). The mRNA expression of IL-1β appeared to show a little increase in ADAR1mφ-/- mouse skins after bleomycin treatment while its protein level was completely blocked by ADAR1-/-. This discrepancy was likely because qPCR detection is much more sensitive than Western blot analysis. However, the increase in IL-1β mRNA level was not significant compared to the PBS treated group. Together, these results indicated that ADAR1 promoted sclerosis development by activating the pro-inflammatory macrophages.

### ADAR1 regulated M1 macrophages *via* NF-κB signaling pathway

**Figure 5:**
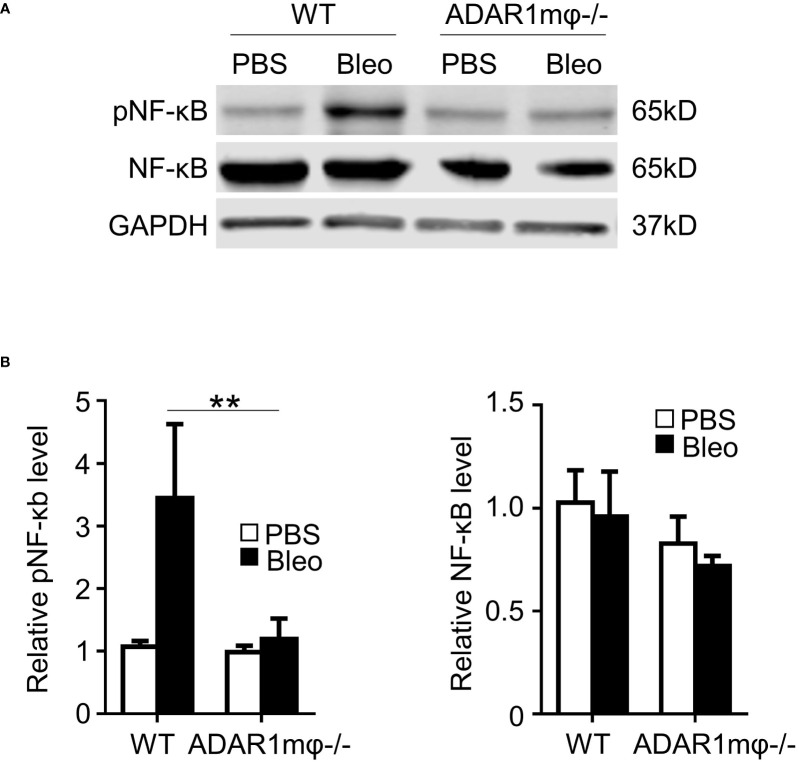
ADAR1 was essential for activation of the NF-κB pathway in M1 macrophages. **(A, B)** ADAR1 deficiency (ADAR1mφ^-/-^) significantly inhibited the bleomycin-induced NF-κB phosphorylation in skin tissues. WT and ADAR1mφ^-/-^ mice were injected s.c. with bleomycin (Bleo, 0.02U) and skin tissues were collected 1d after the treatment. Expression pNF-κB, and NF-κB in the skin tissues was measured by Western blotting **(A)** and quantified by normalizing to GAPDH **(B)** **P < 0.01 vs PBS-treated group in each panel, n = 3. 2-way ANOVA was used for comparison among groups.

It is well-known that NF-κB activation contributes to M1 macrophage activation (2). We hypothesized that ADAR1 activates M1 macrophages by modulating the NF-κB signaling pathway. Thus, we assessed the phosphorylation of NF-κB (p65) in bleomycin-induced skin sclerosis. In agreement with the expression of iNOS and IL-1β (**Figure 4**), bleomycin significantly augmented the NF-κB phosphorylation in WT skin tissues (**Figures 5A, B**) without altering the NF-kB protein expression. However, ADAR1mφ-/- significantly blocked the NF-kB activation. There data indicates that NF-kB signaling plays a critical role in ADAR1 regulated in macrophage activation in Ssc.

### IL-1β is required for ADAR1-mediated SSc

**Figure 6:**
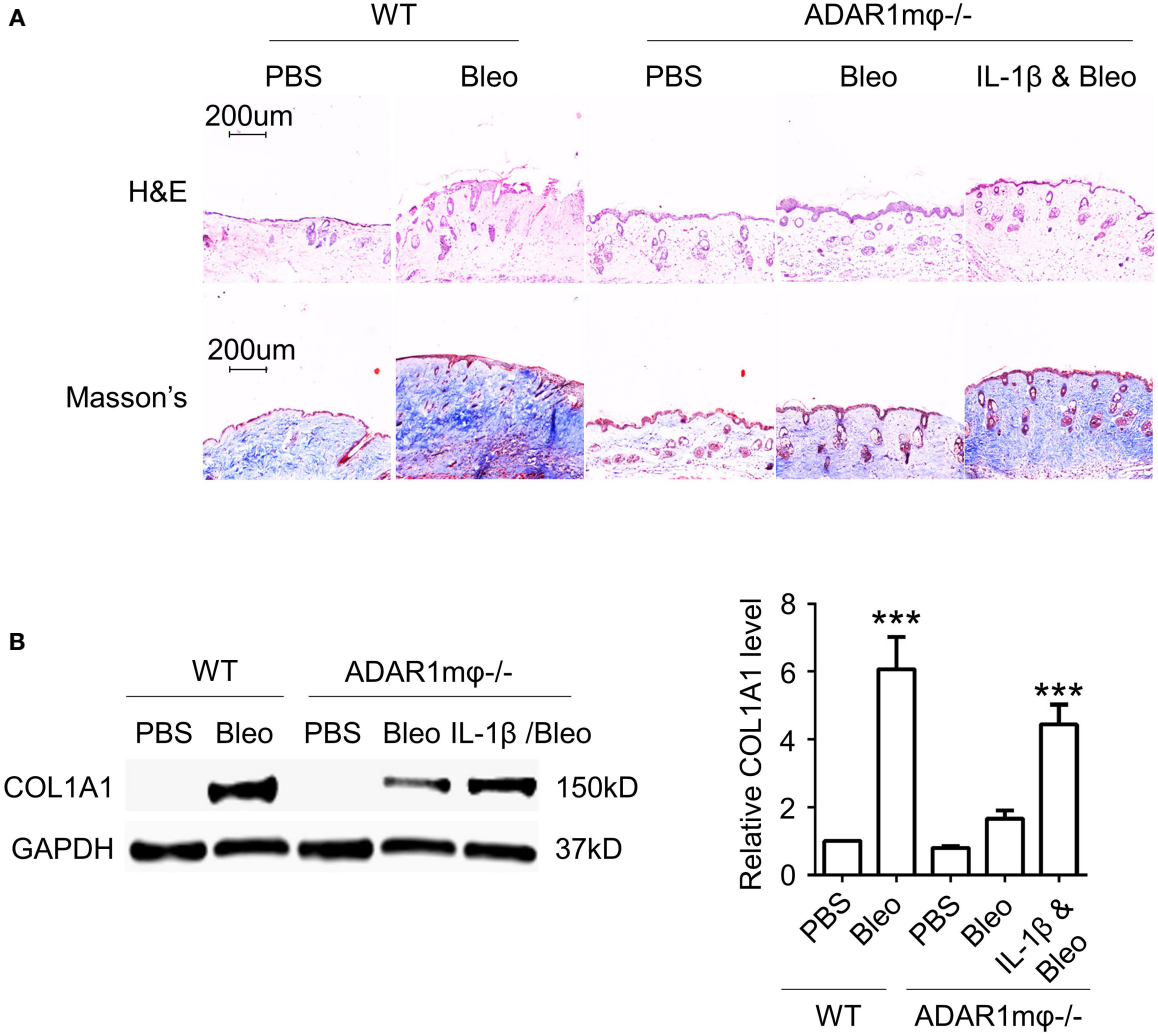
IL-1β is required for ADAR1 mediated SSc. Mice were injected s.c. IL-1β into ADAR1mφ-/- mice followed by vehicle (PBS) or bleomycin (Bleo) treatment, and skin tissues were collected 28d after the treatment. **(A)** IL-1β deteriorated the ADAR1mφ^-/-^ induced skin fibrosis relief, as shown by Masson’s trichrome staining. H&E staining showed skin structure. 20x power of objective lens was used to acquire images. **(B)** IL-1β exacerbated the ADAR1mφ^-/-^ blocked COL1A1 expression in skin tissues, as determined by Western blotting. Col1a1 protein levels were quantified by normalizing to GAPDH. ***P < 0.001, n = 3. 2-way ANOVA was used for comparison among groups.

To determine whether IL-1β is responsible for ADAR1-mediated sclerosis, we injected IL-1β into ADAR1mφ-/- mice followed by bleomycin treatment. As shown in **Figure 6**, adoptive injection of IL-1β deteriorated the relief in skin fibrosis in ADAR1mφ-/- mice treated with bleomycin, as evidenced by the marked increase in skin thickness and collagen deposition/protein expression that were attenuated in ADAR1mφ-/- mice (**Figures 6A, B**). These data demonstrated that ADAR1 regulates SSc development through promoting the inflammatory macrophages to secret IL-1β.

## Discussion

As a chronic autoimmune disorder, SSc is characterized by diffuse fibrosis in the skin, joints, and internal organs (e.g., lungs and kidneys) (37, 38). Accumulation of pro-inflammatory macrophages is an early event essential for SSc development (39, 40). However, the pathophysiological mechanism underlying the role of macrophages in SSc is incompletely understood. Here, we discover that ADAR1 is a novel protein factor essential for SSc pathogenesis because ADAR1 deficiency significantly ameliorated the bleomycin-induced sclerosis. It appears that ADAR1 promotes classic macrophage activation, leading to SSc development. ADAR1 is prominently induced at the initial stage of SSc, concomitant with macrophage accumulation in the skin. More importantly, loss of ADAR1 in macrophages remarkably inhibits the skin and lung fibrosis pathogenesis along with reduction in dermis thickness, lung structure damage, collagen tissue deposition and production. Our mechanistic data indicate that ADAR1 promotes the classic activation of macrophage and inflammatory response through activating NF-κB signaling pathway.

ADAR1 appears to promote sclerosis by stimulating macrophage inflammatory response. We demonstrated that ADAR1 deficiency in macrophages significantly reduces the expression of iNOS and IL-1β in skin tissues. IL-1β has been shown to play a vital role in promoting fibrosis and SSc (35, 36). IL-1β expression level is significantly up-regulated in the serum, bronchoalveolar lavage fluid, and the skin lesions of SSc patients (41). IL-1β deficiency significant attenuates the pulmonary fibrosis induced by bleomycin in mice through relieving fibroblast–myofibroblast differentiation and the myofibroblasts longevity, which are also the critical events in skin fibrosis in SSc patients (42, 43). Our data demonstrated that IL-1β is also critically important for ADAR1-mediated SSc development, as supplementation of IL-1β significantly reverses the reduction of skin fibrosis observed in ADAR1mφ-/- mice treated with bleomycin. It is possible that ADAR1 also mediates fibroblast–myofibroblast differentiation or the myofibroblasts longevity to promote SSc through regulating IL-1β expression. However, this would require extensive future investigation.

ADAR1 participates in regulating innate immune response by both editing-dependent and -independent mechanisms (44). RNA editing of target proteins is the primary function of ADAR1. Additional editing-dependent mechanism of ADAR1 has been proposed to affect mRNA maturation through splicing pre-mRNA (45). Recently, an increasing number of studies have revealed RNA editing-independent roles of ADAR1 in regulating miRNA biogenesis by either acting as an RNA-binding protein or forming complexes with other proteins (22, 46, 47). Therefore, ADAR1 could induce M1 macrophage activation through RNA editing or non-editing mechanisms. The mechanisms by which ADAR1 regulates macrophage polarization is a subject for future investigation.

Although the role of ADAR1 in macrophages and inflammatory response remains controversial, our current results clearly demonstrated that ADAR1 is indispensable for bleomycin-induced classical macrophage activation and its subsequent inflammatory response. This outcome is validated *in vivo* in bleomycin-induced SSc mouse model. The discrepancy of the ADAR1 functions in macrophage studies including ours is likely due to the different approaches blocking ADAR1 expression and/or the use of different source of macrophages. Nonetheless, our studies reveal a crucial role for ADAR1 in the development of SSc. ADAR1 promotes M1 macrophage activation and regulates the expression of inflammatory mediators through modulating NF-κB signaling. Therefore, targeting ADAR1 could be a novel therapeutic strategy in treating systemic sclerosis.

## Data availability statement

The original contributions presented in the study are included in the article/Supplementary Material. Further inquiries can be directed to the corresponding authors.

## Ethics statement

The animal study was reviewed and approved by The Institutional Animal Care and Use Committee of Xi’an Jiaotong University, Xi’an Center for Disease Control.

## Author contributions

CS and S-YC conceived the study and designed the experiments. CS and DC performed the research and analyzed the data. CS and S-YC wrote the paper. All authors contributed to the article and approved the submitted version.

## Funding

This work was supported by grants from Natural Science Foundation of China (82071828, CS), National Institutes of Health (HL119053 and HL147313, S-YC), China Postdoctoral Science Foundation (2021M692559, CS), and Early Career Research Start-up Plan of Xi’an Jiaotong University, Central University Basic Research Fund (xxj032022001, CS).

## Conflict of interest

The authors declare that the research was conducted in the absence of any commercial or financial relationships that could be construed as a potential conflict of interest.

## Publisher’s note

All claims expressed in this article are solely those of the authors and do not necessarily represent those of their affiliated organizations, or those of the publisher, the editors and the reviewers. Any product that may be evaluated in this article, or claim that may be made by its manufacturer, is not guaranteed or endorsed by the publisher.

## References

[1] Almeida I Faria R Vita P Vasconcelos C. Systemic sclerosis refractory disease: from the skin to the heart. Autoimmun Rev (2011) 10(11):693–701. doi: 10.1016/j.autrev.2011.04.025 21575745

[2] Sun C Chen SY. RGC32 promotes bleomycin-induced systemic sclerosis in a murine disease model by modulating classically activated macrophage function. J Immunol (2018) 200(8):2777–85. doi: 10.4049/jimmunol.1701542 PMC5893351 29507108

[3] Taniguchi T Asano Y Akamata K Noda S Takahashi T Ichimura Y. Fibrosis, vascular activation, and immune abnormalities resembling systemic sclerosis in bleomycin-treated fli-1-haploinsufficient mice. Arthritis Rheumatol (2015) 67(2):517–26. doi: 10.1002/art.38948 PMC4342755 25385187

[4] Papadimitriou TI Van Caam A van der Kraan PM Thurlings RM. Therapeutic options for systemic sclerosis: Current and future perspectives in tackling immune-mediated fibrosis. Biomedicines (2022) 10(2):316. doi: 10.3390/biomedicines10020316 PMC8869277 35203525

[5] Lakos G Melichian D Wu M Varga J. Increased bleomycin-induced skin fibrosis in mice lacking the Th1-specific transcription factor T-bet. Pathobiology (2006) 73(5):224–37. doi: 10.1159/000098208 17314493

[6] Yamamoto T Nishioka K. Animal model of sclerotic skin. VI: Evaluation of bleomycin-induced skin sclerosis in nude mice. Arch Dermatol Res (2004) 295(10):453–6. doi: 10.1007/s00403-003-0439-y 14673598

[7] Helene M Lake-Bullock V Zhu J Hao H Cohen DA Kaplan AM. T Cell independence of bleomycin-induced pulmonary fibrosis. J Leukoc Biol (1999) 65(2):187–95. doi: 10.1002/jlb.65.2.187 10088601

[8] Yunna C Mengru H Lei W Weidong C. Macrophage M1/M2 polarization. Eur J Pharmacol (2020) 877:173090. doi: 10.1016/j.ejphar.2020.173090 32234529

[9] Weichhart T Hengstschlager M Linke M. Regulation of innate immune cell function by mTOR. Nat Rev Immunol (2015) 15(10):599–614. doi: 10.1038/nri3901 26403194 PMC6095456

[10] Lawrence T Bebien M Liu GY Nizet V Karin M. IKKalpha limits macrophage NF-kappaB activation and contributes to the resolution of inflammation. Nature (2005) 434(7037):1138–43. doi: 10.1038/nature03491 15858576

[11] Xue D Tabib T Morse C Yang Y Domsic RT Khanna D. Expansion of fcgamma receptor IIIa-positive macrophages, ficolin 1-positive monocyte-derived dendritic cells, and plasmacytoid dendritic cells associated with severe skin disease in systemic sclerosis. Arthritis Rheumatol (2022) 74(2):329–41. doi: 10.1002/art.41813 PMC8626521 34042322

[12] Salim PH Jobim M Bredemeier M Chies JA Brenol JC Jobim LF. Interleukin-10 gene promoter and NFKB1 promoter insertion/deletion polymorphisms in systemic sclerosis. Scand J Immunol (2013) 77(2):162–8. doi: 10.1111/sji.12020 23237063

[13] Ciechomska M Wojtas B Swacha M Olesinska M Benes V Maslinski W. Global miRNA and mRNA expression profiles identify miRNA-26a-2-3p-dependent repression of IFN signature in systemic sclerosis human monocytes. Eur J Immunol (2020) 50(7):1057–66. doi: 10.1002/eji.201948428 32087087

[14] Song G Yang R Zhang Q Chen L Huang D Zeng J. TGF-beta secretion by M2 macrophages induces glial scar formation by activating astrocytes *In vitro* . J Mol Neurosci (2019) 69(2):324–32. doi: 10.1007/s12031-019-01361-5 31327154

[15] Furukawa S Moriyama M Tanaka A Maehara T Tsuboi H Iizuka M. Preferential M2 macrophages contribute to fibrosis in IgG4-related dacryoadenitis and sialoadenitis, so-called mikulicz's disease. Clin Immunol (2015) 156(1):9–18. doi: 10.1016/j.clim.2014.10.008 25450336

[16] Trojanowska M. Role of PDGF in fibrotic diseases and systemic sclerosis. Rheumatol (Oxford) (2008) 47(Suppl 5):v2–4. doi: 10.1093/rheumatology/ken265 18784131

[17] Licht K Jantsch MF. Rapid and dynamic transcriptome regulation by RNA editing and RNA modifications. J Cell Biol (2016) 213(1):15–22. doi: 10.1083/jcb.201511041 27044895 PMC4828693

[18] Wu M Jin M Cao X Qian K Zhao L. RNA Editing enzyme adenosine deaminases acting on RNA 1 deficiency increases the sensitivity of non-small cell lung cancer cells to anlotinib by regulating CX3CR1-fractalkine expression. Drug Dev Res (2022) 83(2):328–38. doi: 10.1002/ddr.21861 34319598

[19] Ishizuka JJ Manguso RT Cheruiyot CK Bi K Panda A Iracheta-Vellve A. Loss of ADAR1 in tumours overcomes resistance to immune checkpoint blockade. Nature (2019) 565(7737):43–8. doi: 10.1038/s41586-018-0768-9 PMC7241251 30559380

[20] Song C Sakurai M Shiromoto Y Nishikura K. Functions of the RNA editing enzyme ADAR1 and their relevance to human diseases. Genes (Basel) (2016) 7(12):129. doi: 10.3390/genes7120129 27999332 PMC5192505

[21] Nishikura K. A-to-I editing of coding and non-coding RNAs by ADARs. Nat Rev Mol Cell Biol (2016) 17(2):83–96. doi: 10.1038/nrm.2015.4 26648264 PMC4824625

[22] Nemlich Y Baruch EN Besser MJ Shoshan E Bar-Eli M Anafi L. ADAR1-mediated regulation of melanoma invasion. Nat Commun (2018) 9(1):2154. doi: 10.1038/s41467-018-04600-2 29855470 PMC5981216

[23] Bahn JH Ahn J Lin X Zhang Q Lee JH Civelek M. Genomic analysis of ADAR1 binding and its involvement in multiple RNA processing pathways. Nat Commun (2015) 6:6355. doi: 10.1038/ncomms7355 25751603 PMC4355961

[24] Vlachogiannis NI Tual-Chalot S Zormpas E Bonini F Ntouros PA Pappa M. Adenosine-to-inosine RNA editing contributes to type I interferon responses in systemic sclerosis. J Autoimmun (2021) 125:102755. doi: 10.1016/j.jaut.2021.102755 34857436 PMC8713031

[25] Yang JH Luo X Nie Y Su Y Zhao Q Kabir K. Widespread inosine-containing mRNA in lymphocytes regulated by ADAR1 in response to inflammation. Immunology (2003) 109(1):15–23. doi: 10.1046/j.1365-2567.2003.01598.x 12709013 PMC1782949

[26] Wu Y Wang H Zhang J Ma X Meng J Li Y. Adenosine deaminase that acts on RNA 1 p150 in alveolar macrophage is involved in LPS-induced lung injury. Shock (2009) 31(4):410–15. doi: 10.1097/SHK.0b013e31817c1068 18520702

[27] Ben-Shoshan SO Kagan P Sultan M Barabash Z Dor C Jacob-Hirsch J. ADAR1 deletion induces NFkappaB and interferon signaling dependent liver inflammation and fibrosis. RNA Biol (2017) 14(5):587–602. doi: 10.1080/15476286.2016.1203501 27362366 PMC5449086

[28] Liu S Xie J Zhao B Hu X Li X Zhang B. ADAR1 prevents small intestinal injury from inflammation in a murine model of sepsis. Cytokine (2018) 104:30–7. doi: 10.1016/j.cyto.2018.01.020 29414324

[29] Wang G Wang H Singh S Zhou P Yang S Wang Y. ADAR1 prevents liver injury from inflammation and suppresses interferon production in hepatocytes. Am J Pathol (2015) 185(12):3224–37. doi: 10.1016/j.ajpath.2015.08.002 PMC4729276 26453800

[30] Cavassani KA Ishii M Wen H Schaller MA Lincoln PM Lukacs NW. TLR3 is an endogenous sensor of tissue necrosis during acute inflammatory events. J Exp Med (2008) 205(11):2609–21. doi: 10.1084/jem.20081370 PMC2571935 18838547

[31] Chu Z Sun C Sun L Feng C Yang F Xu Y. Primed macrophages directly and specifically reject allografts. Cell Mol Immunol (2020) 17(3):237–46. doi: 10.1038/s41423-019-0226-0 PMC7052205 30948792

[32] Toledo DM Pioli PA. Macrophages in systemic sclerosis: Novel insights and therapeutic implications. Curr Rheumatol Rep (2019) 21(7):31. doi: 10.1007/s11926-019-0831-z 31123840 PMC7444604

[33] Morales-Cardenas A Perez-Madrid C Arias L Ojeda P Mahecha MP Rojas-Villarraga A. Pulmonary involvement in systemic sclerosis. Autoimmun Rev (2016) 15(11):1094–108. doi: 10.1016/j.autrev.2016.07.025 27497912

[34] Carvalheiro T Affandi AJ Malvar-Fernandez B Dullemond I Cossu M Ottria A. Induction of inflammation and fibrosis by semaphorin 4A in systemic sclerosis. Arthritis Rheumatol (2019) 71(10):1711–22. doi: 10.1002/art.40915 PMC6790618 31012544

[35] De Luca G Cavalli G Campochiaro C Bruni C Tomelleri A Dagna L. Interleukin-1 and systemic sclerosis: Getting to the heart of cardiac involvement. Front Immunol (2021) 12:653950. doi: 10.3389/fimmu.2021.653950 33833766 PMC8021854

[36] Maria ATJ Rozier P Fonteneau G Sutra T Maumus M Toupet K. iNOS activity is required for the therapeutic effect of mesenchymal stem cells in experimental systemic sclerosis. Front Immunol (2018) 9:3056. doi: 10.3389/fimmu.2018.03056 30622540 PMC6308989

[37] Korman BD Criswell LA. Recent advances in the genetics of systemic sclerosis: toward biological and clinical significance. Curr Rheumatol Rep (2015) 17(3):21. doi: 10.1007/s11926-014-0484-x 25777745 PMC4361757

[38] Lefevre G Dauchet L Hachulla E Montani D Sobanski V Lambert M. Survival and prognostic factors in systemic sclerosis-associated pulmonary hypertension: a systematic review and meta-analysis. Arthritis Rheumatol (2013) 65(9):2412–23. doi: 10.1002/art.38029 23740572

[39] Bhandari R Ball MS Martyanov V Popovich D Schaafsma E Han S. Profibrotic activation of human macrophages in systemic sclerosis. Arthritis Rheumatol (2020) 72(7):1160–9. doi: 10.1002/art.41243 PMC7329566 32134204

[40] Lescoat A Lecureur V Varga J. Contribution of monocytes and macrophages to the pathogenesis of systemic sclerosis: recent insights and therapeutic implications. Curr Opin Rheumatol (2021) 33(6):463–70. doi: 10.1097/BOR.0000000000000835 34506339

[41] Martinez-Godinez MA Cruz-Dominguez MP Jara LJ Dominguez-Lopez A Jarillo-Luna RA Vera-Lastra O. Expression of NLRP3 inflammasome, cytokines and vascular mediators in the skin of systemic sclerosis patients. Isr Med Assoc J (2015) 17(1):5–10. 25739168

[42] Kolb M Margetts PJ Anthony DC Pitossi F Gauldie J. Transient expression of IL-1beta induces acute lung injury and chronic repair leading to pulmonary fibrosis. J Clin Invest (2001) 107(12):1529–36. doi: 10.1172/JCI12568 PMC200196 11413160

[43] Xu D Mu R Wei X. The roles of IL-1 family cytokines in the pathogenesis of systemic sclerosis. Front Immunol (2019) 10:2025. doi: 10.3389/fimmu.2019.02025 31572353 PMC6753625

[44] Heraud-Farlow JE Walkley CR. The role of RNA editing by ADAR1 in prevention of innate immune sensing of self-RNA. J Mol Med (Berl) (2016) 94(10):1095–102. doi: 10.1007/s00109-016-1416-1 27044320

[45] Fei J Cui XB Wang JN Dong K Chen SY. ADAR1-mediated RNA editing, a novel mechanism controlling phenotypic modulation of vascular smooth muscle cells. Circ Res (2016) 119(3):463–9. doi: 10.1161/CIRCRESAHA.116.309003 PMC4961590 27199464

[46] Chen T Xiang JF Zhu S Chen S Yin QF Zhang XO. ADAR1 is required for differentiation and neural induction by regulating microRNA processing in a catalytically independent manner. Cell Res (2015) 25(4):459–76. doi: 10.1038/cr.2015.24 PMC4387555 25708366

[47] Ota H Sakurai M Gupta R Valente L Wulff BE Ariyoshi K. ADAR1 forms a complex with dicer to promote microRNA processing and RNA-induced gene silencing. Cell (2013) 153(3):575–89. doi: 10.1016/j.cell.2013.03.024 PMC3651894 23622242

